# Lipid levels and risk of venous thrombosis: results from the MEGA-study

**DOI:** 10.1007/s10654-017-0251-1

**Published:** 2017-05-24

**Authors:** Vânia M. Morelli, Willem M. Lijfering, Mettine H. A. Bos, Frits R. Rosendaal, Suzanne C. Cannegieter

**Affiliations:** 1 Department of Clinical Epidemiology, Leiden University Medical Center, PO Box 9600, 2300 RC Leiden, The Netherlands; 2 Department of Clinical and Experimental Oncology, Federal University of São Paulo, São Paulo, Brazil; 3 Einthoven Laboratory for Experimental Vascular Medicine, Leiden University Medical Center, Leiden, The Netherlands; 4 Department of Internal Medicine, Section of Thrombosis and Haemostasis, Leiden University Medical Centre, Leiden, The Netherlands

**Keywords:** Epidemiology, Lipids, Lipoproteins, Apolipoproteins, Risk factors, Venous thrombosis

## Abstract

The relationship between lipid levels and risk of venous thrombosis is not well established. We aimed to assess the association between several lipids and risk of venous thrombosis using data from a population-based case–control study, and to evaluate the underlying mechanism, considering confounding by common risk factors and mediation via hemostatic factors and C-reactive protein. From the Multiple Environmental and Genetic Assessment of risk factors for venous thrombosis (MEGA) study, 2234 patients with a first venous thrombosis and 2873 controls were included. Percentile categories of total/low-density lipoprotein/high-density lipoprotein cholesterol, triglycerides, and apolipoproteins B and A1 were established in controls (<10th, 10th–25th, 25th–75th [reference], 75th–90th, >90th percentile). In age- and sex-adjusted models, decreasing levels of apolipoproteins B and A1 were dose-dependently associated with increased thrombosis risk, with odds ratios of 1.35 (95% confidence interval 1.12–1.62) and 1.50 (95% confidence interval 1.25–1.79) for the lowest category versus the reference category, respectively. The dose–response relation remained with further adjustment for body mass index, estrogen use, statin use, and diabetes. Although apolipoproteins B and A1 were associated with several hemostatic factors and C-reactive protein, none explained the increased risk in mediation analyses. The other lipids were not associated with venous thrombosis risk. In conclusion, decreasing levels of apolipoproteins B and A1 were associated with increased risk of venous thrombosis. Our findings are consistent with experimental data on the anticoagulant properties of apolipoproteins B and A1. These findings need to be confirmed and the underlying mechanism further investigated.

**Electronic supplementary material:**

The online version of this article (doi:10.1007/s10654-017-0251-1) contains supplementary material, which is available to authorized users.

## Introduction

Venous thrombosis and arterial cardiovascular disease have been traditionally regarded as separate diseases with distinct causes and treatment. However, several studies in the past decade have shown that patients with venous thrombosis (i.e., deep vein thrombosis or pulmonary embolism) have an increased risk of subsequent arterial disease [1]. As lipid levels can be modulated by lifestyle intervention and drug therapy [2], the potential association between lipids and venous thrombosis and the related pathophysiology is a relevant clinical issue worth pursuing. Indeed, previous data have shown that lipid-lowering drugs (statins, most notably rosuvastatin) are associated with a decreased risk of venous thrombosis [3–5], which might indicate a possible role for lipids in the pathophysiology of venous thrombosis. However, whether lipids are associated with venous thrombosis is not known in detail due to controversial results among epidemiological studies [6–13].

**Fig. 1:**
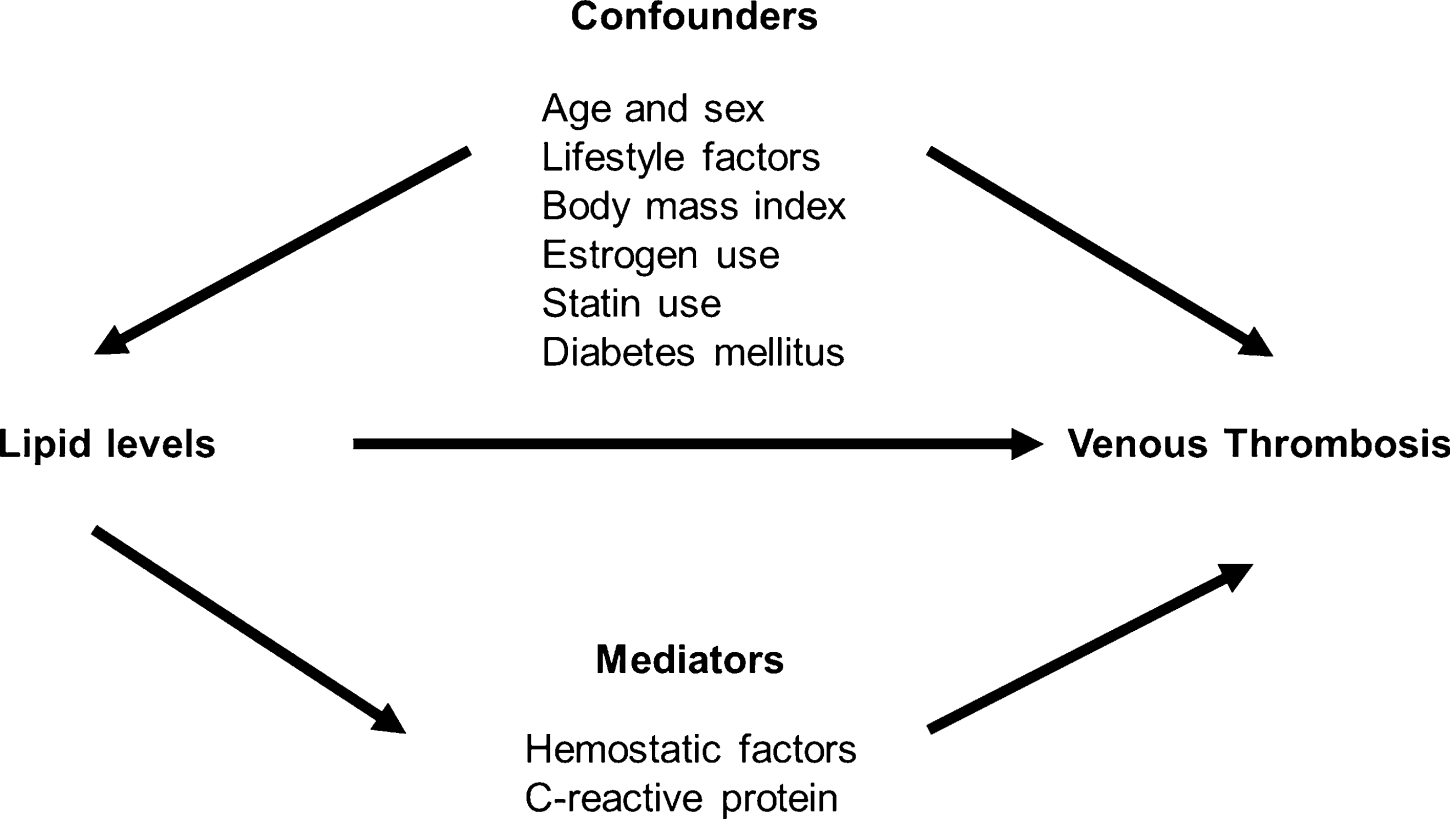
Causal diagram of the association between lipid levels and venous thrombosis

The association between lipid levels and venous thrombosis might be explained by common factors that are related to both lipids and risk of venous thrombosis (confounders), such as age [14–16], sex [11, 14, 15], lifestyle [2, 17], body mass index (BMI) [2, 15, 16], estrogen use [15, 16, 18], statin use [3–5, 19], and diabetes mellitus [2, 20] (Fig. 1). If causal, the association could be explained by factors that are a consequence of lipid properties on hemostasis [21] and inflammation [22] (mediators in the causal pathway) (Fig. 1). These might increase the risk of venous thrombosis and could include changes in levels of hemostatic factors [16] and C-reactive protein (CRP) [10].

Therefore, the aim of our study was to assess the association between lipid levels and risk of venous thrombosis, and to evaluate the underlying mechanism, with particular attention to confounding and mediation via hemostatic factors and CRP. For this purpose, we used data of a large, population-based case–control study on the etiology of venous thrombosis [Multiple Environmental and Genetic Assessment of risk factors for venous thrombosis (MEGA) study]. In the present analysis, we included the major lipid analytes, i.e., total cholesterol (TC), low-density lipoprotein cholesterol (LDL-C), high-density lipoprotein cholesterol (HDL-C), and triglycerides. We also analysed apolipoprotein A1 (apo A1), which is the major protein component of HDL-C [23], and apolipoprotein B (apo B), which is the protein component of the very low-density/low-density lipoprotein spectrum (apo B-containing lipoproteins) [23].

## Methods

### Study design

**Fig. 2:**
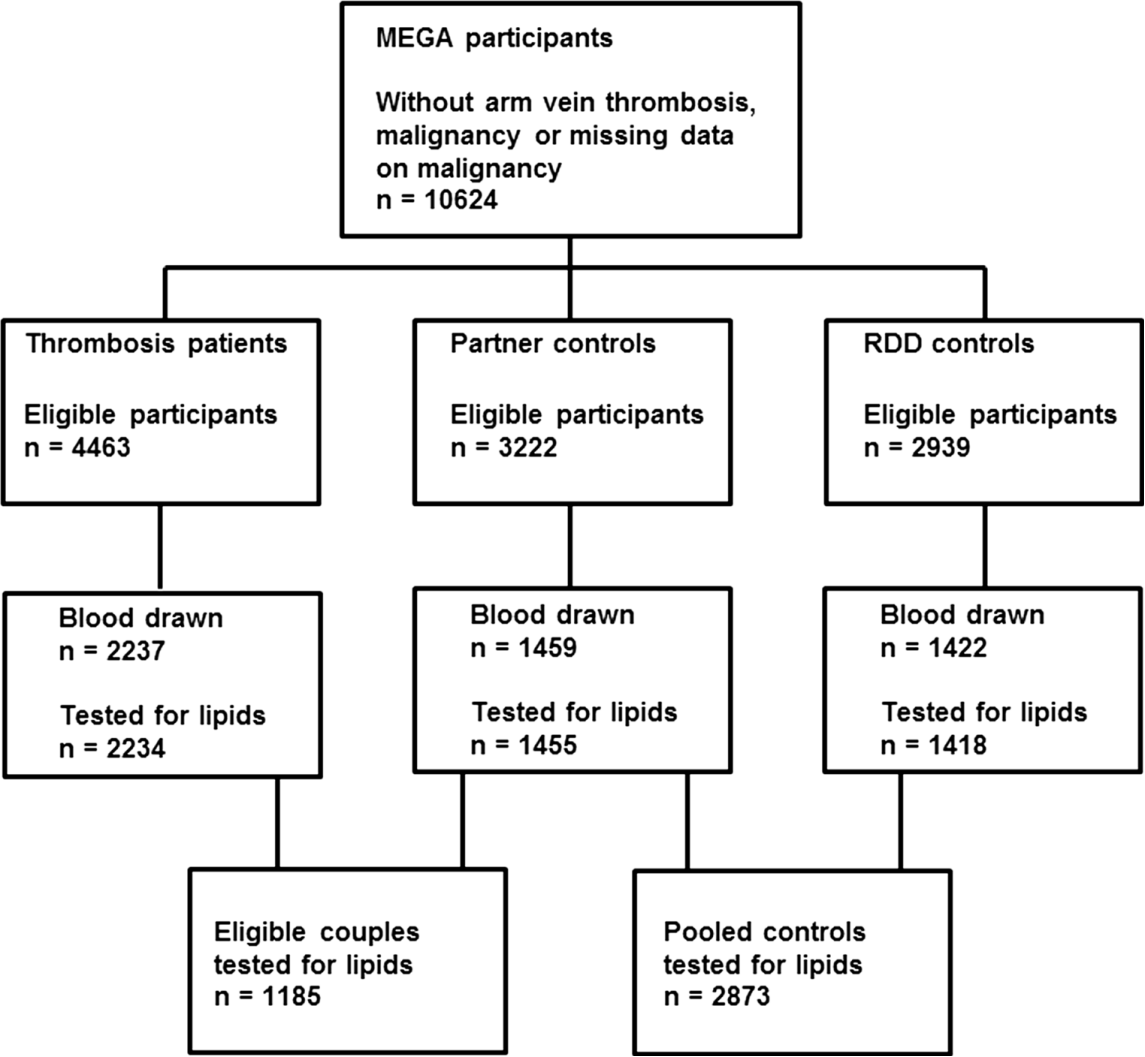
Flow chart of the Multiple Environmental and Genetic Assessment of risk factors for venous thrombosis (MEGA) study. *RDD* random-digit dialing

Between March 1999 and September 2004, consecutive patients aged 18–70 years with a first objectively confirmed deep vein thrombosis or pulmonary embolism were included in the MEGA study from 6 anticoagulation clinics in the Netherlands [24]. For the current analysis, patients with arm vein thrombosis and participants with active or previous history of malignancy within 5 years before the index date, and participants with missing data on malignancy were excluded. In the MEGA study, blood sampling was determined by calendar time, i.e., for logistic reasons participants were asked to provide blood samples up to June 2002 only [24]. Of the 4463 patients eligible for this study, 2237 provided blood samples (Fig. 2). Controls were included from 2 sources: partners of patients and subjects reached by random digit dialing (RDD) [24] (Fig. 2). Of the 3222 eligible partners (aged 18–70 years, and without venous thrombosis), 1459 provided blood. Of the 2939 eligible RDD controls (frequency matched for age and sex to the patients, and without venous thrombosis), 1422 provided blood. This study was approved by the Ethics Committee of the Leiden University Medical Center, and written informed consent was obtained from all participants. The MEGA study has been conducted according to the principles expressed in the Declaration of Helsinki and described in detail elsewhere [24].

### Data collection and blood sampling

All participants were asked to complete a questionnaire on many potential risk factors for venous thrombosis [24]. Of interest for this analysis are the items on body weight and height, lifestyle, estrogen- and statin-use, and self-reported diabetes. BMI was calculated by dividing weight (kg) by height squared (m^2^). A BMI between 18.5 and 25 kg/m^2^ was defined as normal, between 25 and 30 kg/m^2^ as overweight and >30 kg/m^2^ as obesity. The index date was defined as the date of diagnosis of venous thrombosis for patients and their partners, and the date of completing the questionnaire for RDD controls. At least 3 months after discontinuation of anticoagulation, or during anticoagulant therapy in patients who continued this therapy for more than 1 year, patients and controls visited the anticoagulation clinic for an interview and blood sampling.

### Laboratory measurements

Lipids were measured on stored (−80 °C) and previously unthawed fasting serum samples. TC and triglycerides were measured by a colorimetric method (CHOD-PAP for TC and GPO-PAP for triglycerides) on a Modular P analyser (Roche Diagnostics, Mannheim, Germany). HDL-C was measured by a direct method based on the Kyowa Medex reaction principle using polyethylene glycol (PEG)-modified enzymes (Roche Diagnostics, Mannheim, Germany). Apo A1 and apo B were measured by immunoturbidimetric assays on a Cobas Integra analyzer (Roche Diagnostics, Mannheim, Germany). LDL-C levels were estimated using the Friedewald formula [LDL-C = TC − HDL-C − (triglycerides/2.2) for mmol/L] [25], and when triglycerides exceeded 4.52 mmol/L, LDL-C was not estimated. The natural anticoagulants (antithrombin, protein C, and total protein S), the procoagulant factors (fibrinogen, factors II, VII, VIII, IX, X and XI, and von Willebrand factor), clot lysis time, and CRP levels were determined according to methods previously described [26, 27]. Total tissue factor pathway inhibitor (TFPI) activity levels were assessed in citrated plasma by measuring TFPI inhibition of the catalytic tissue factor (TF)-factor VIIa (FVIIa) complex using the Actichrome TFPI activity assay (Sekisui Diagnostics, Stamford, CT, USA); one unit of TFPI activity corresponds to 55 ng/ml plasma TFPI. All laboratory analyses were performed without knowledge of whether the sample was from a patient or a control subject.

### Statistical analyses

#### Demographic and clinical characteristics related to lipid levels in controls

To obtain insight in potential confounding variables, we estimated in the pooled control group (partner and RDD controls) mean differences and their 95% confidence intervals (CIs) in lipid levels by linear regression in relation to age (50–70 years vs. 18–50 years [reference]), sex (women vs. men [reference]), BMI (overweight/obesity vs. normal weight [reference]), self-reported diabetes (yes vs. no [reference]), estrogen use at blood sampling (users vs. nonusers [reference]), and statin use (users vs. nonusers [reference]). All lipids were normally distributed, with the exception of triglycerides (right-skewed distribution), which levels were log-transformed. In our regression models, each lipid was entered as the dependent variable, and the demographic or clinical characteristics (i.e., age, sex, BMI, self-reported diabetes, estrogen use at blood sampling, and statin use) were the independent variables. The resulting regression coefficient (β) for a clinical or demographic characteristic indicated the mean difference in lipid levels between the reference and the other category of that particular characteristic. When applicable, mean differences and their 95% CIs were adjusted for age (continuous) and sex, and further for the other aforementioned characteristics.

#### Lipid levels and risk of venous thrombosis

Lipid categories were defined according to the values measured in the pooled control group (<10th, 10th–25th, 25th–75th, 75th–90th, and >90th percentile). Age- and sex-adjusted odds ratios (OR) and their 95% CIs were calculated as estimates of the relative risk of venous thrombosis for the different lipid categories in comparison with the reference category (25th–75th percentiles) by unconditional logistic regression. We further adjusted for other potential confounders to assess whether an increased thrombosis risk could be explained by these factors i.e.: estrogen use at blood sampling (dichotomous value), BMI (continuous values), statin use (dichotomous value), and self-reported diabetes (dichotomous value).

Dyslipidemia may be related to lifestyle [2], which may affect venous thrombosis risk [17], and therefore lifestyle may act as another confounder. Such behavior is not easily measured and adjusted for. Partners of patients are likely to resemble the patients in lifestyle, and therefore we performed an additional 1:1 matched analysis by conditional logistic regression which adjusts for associations within matched couples. This method fully takes matching into account, with adjustment for all unmeasured factors for which couples tend to be similar (e.g., socioeconomic class) [28]. In this analysis, all aforementioned potential confounders were additionally adjusted for as covariates. Although using partners as controls results in most controls having the opposite sex as their matched case, one can adjust for sex in a partner-matched case–control study by adding sex to the model [28].

#### Mediation analyses

In case an association between lipid levels and venous thrombosis is present, a mediation analysis is useful to assess whether this association could be explained by factors related to hemostasis and inflammation. First, we investigated whether lipid levels were associated with changes in hemostatic factor and CRP levels in the general population by evaluating the pooled control group. For this purpose, we used linear regression to estimate mean differences and their 95% CIs in levels of hemostatic factors and CRP for the 10th–25th, 25th–75th, 75th–90th, and >90th percentile categories of lipid levels in comparison with the reference category (<10th percentile). Because CRP levels showed a distribution skewed to the right, a log-transformation was applied for this variable. In our regression models, CRP (log-transformed) or each of the hemostatic factors studied was entered as the dependent variable, and the aforementioned lipid categories were the independent variables. The resulting β coefficient for a lipid category indicated the mean difference in levels of hemostatic factors or CRP between that particular category and the reference. Mean differences and their 95% CIs were adjusted for age, sex, estrogen use at blood sampling, BMI, statin use, and self-reported diabetes. We further used linear regression to estimate the increase or decrease in levels of hemostatic factors or CRP (log-transformed) for every 1 unit increase in lipid levels after adjustment for the same potential confounders. Controls (n = 27) who used vitamin K antagonists at the time of blood draw were excluded from the analyses of the vitamin K-dependent factors.

Second, we repeated the earlier logistic regression analyses, at this time including factors associated with lipid levels. Hemostatic factors and CRP (log-transformed) were introduced in the logistic regression model as continuous variables. Patients (n = 271) and controls (n = 27) using vitamin K antagonists at the time of blood draw were excluded if vitamin K-dependent factors were added to the model.

All statistical analyses were performed with SPSS for Windows, release 20.0 (SPSS Inc, Chicago, IL).

## Results

### Clinical characteristics

Assessment of lipids was successful for over 99% of the eligible participants, with a total of 2234 patients, 1455 partner and 1418 RDD controls (Fig. 2). Of the 1455 partners, 1185 were matched with patients. Table 1 shows the baseline characteristics of the participants. There were no substantial age and sex differences between patients and controls. BMI was higher in patients than in RDD controls and it was virtually the same as in partners. Patients used hormones more often at the index date than controls, and controls were more likely to use hormones at blood sampling, as most patients discontinued hormone use after venous thrombosis. The percentage of self-reported diabetes was virtually the same among participants, and controls used statins more often than patients. The median time between the thrombotic event and blood collection was 10.0 months (interquartile range 8.3–12.4 months). Supplementary Table 1 (Online Resource) shows no substantial differences in the baseline characteristics of all participants compared with those who provided blood samples, indicating that the tested individuals were representative of the whole MEGA group.

**Table 1:** Baseline characteristics

	Patients n = 2234	Partners n = 1455	RDD n = 1418	Partners + RDD n = 2873
Age, years	49 (26–67)	51 (29–66)	48 (24–67)	50 (27–67)
Women, n (%)	1217 (55)	746 (51)	763 (54)	1509 (53)
BMI (kg/m^2^)	26 (20–35)	26 (20–33)	25 (20–32)	25 (20–33)
Estrogen use at index date, n (% in women)	726 (61)	179 (25)	227 (30)	406 (28)
Estrogen use at blood sampling, n (% in women)	205 (18)	159 (23)	222 (30)	381 (26)
Statin use, n (%)	72 (3)	75 (5)	108 (8)	183 (6)
Self-reported diabetes, n (%)	72 (3)	46 (3)	41 (3)	87 (3)
*Venous thrombosis*				
DVT, n (%)	1307 (59)	NA	NA	NA
PE ± DVT, n (%)	927 (41)	NA	NA	NA

Continuous variables are shown as median (5th percentile–95th percentile) and categorical variables as number (%)

Data were missing for some participants in some subgroups

*RDD* random-digit dialing, *BMI* body mass index, *DVT* deep vein thrombosis, *PE* pulmonary embolism, *NA* not applicable

### Demographic and clinical characteristics related to lipid levels in controls

Variation in lipid levels according to demographic and clinical characteristics in controls is detailed in Supplementary Table 2 (Online Resource). Lipid levels increased with age, and men (as compared with women) had higher levels of LDL-C, triglycerides, and apo B and lower levels of HDL-C and apo A1. Levels of HDL-C and apo A1 were lower, whereas levels of the other lipids were higher in overweight/obese controls than in those with normal weight. TC, LDL-C, and apo B were, as expected, reduced among statin users. Estrogen users had higher levels of triglycerides, apo B, and apo A1 as compared with nonusers. Except for triglycerides, self-reported diabetes was associated with a reduction in lipid levels.

### Lipid levels and risk of venous thrombosis

Table 2 lists the risk of venous thrombosis for percentile categories of lipid levels. When partners and RDD controls were pooled as the control group, TC, LDL-C, and triglycerides levels were not associated with venous thrombosis in the age- and sex-adjusted model and after full adjustment. In contrast, decreasing apo B levels were associated with an increasing venous thrombosis risk in the pooled control analyses. In the age- and sex-adjusted model, the lowest percentile category of apo B as compared with the reference category resulted in a 1.35-fold (95% CI 1.12–1.62) increased risk of venous thrombosis (Table 2), and the association was strengthened with further adjustment for BMI (OR 1.56, 95% CI 1.28–1.89). Addition of the other potential confounders to the model did not substantially change risk estimates, which increased dose-dependently with the reduction of apo B levels across percentile categories (Table 2).

**Table 2:** Risk of venous thrombosis by percentiles of lipid levels

	Patients (n = 2234)	Controls (n = 2873)	Pooled control analyses		Partner control analyses	
			OR (95% CI)^a^	OR (95% CI)^b^	OR (95%CI)^a^	OR (95% CI)^b^
TC (mmol L^−1^)						
<10th (<4.28)	217 (10)	286 (10)	0.94 (0.78–1.15)	1.04 (0.84–1.29)	1.09 (0.80–1.49)	1.21 (0.87–1.69)
10th–25th (4.28–4.84)	346 (15)	428 (15)	1.02 (0.87–1.21)	1.06 (0.89–1.26)	0.87 (0.69–1.11)	0.98 (0.75–1.27)
25th–75th (4.84–6.30)	1107 (50)	1431 (50)	1 (reference)	1 (reference)	1 (reference)	1 (reference)
75th–90th (6.30–7.04)	332 (15)	441 (15)	0.98 (0.83–1.16)	0.96 (0.81–1.14)	0.74 (0.59–0.94)	0.78 (0.61–1.01)
>90th (>7.04)	232 (10)	287 (10)	1.07 (0.88–1.29)	1.00 (0.82–1.22)	0.80 (0.61–1.06)	0.82 (0.60–1.11)
LDL-C (mmol L^−1^)						
<10th (<2.38)	213 (10)	282 (10)	0.92 (0.76–1.12)	1.09 (0.88–1.35)	1.16 (0.85–1.59)	1.42 (1.01–1.99)
10th–25th (2.38–2.87)	304 (14)	425 (15)	0.88 (0.75–1.04)	0.97 (0.81–1.15)	0.98 (0.75–1.26)	1.05 (0.79–1.39)
25th–75th (2.87–4.17)	1118 (51)	1414 (50)	1 (reference)	1 (reference)	1 (reference)	1 (reference)
75th–90th (4.17–4.85)	342 (15)	425 (15)	1.03 (0.87–1.21)	1.02 (0.86–1.21)	0.92 (0.73–1.15)	0.88 (0.68–1.13)
>90th (>4.85)	228 (10)	282 (10)	1.05 (0.86–1.27)	0.98 (0.80–1.20)	0.93 (0.71–1.22)	0.96 (0.71–1.29)
Triglycerides (mmol L^−1^)						
<10th (<0.79)	224 (10)	273 (10)	1.02 (0.84–1.25)	1.14 (0.92–1.40)	1.26 (0.92–1.72)	1.39 (0.99–1.95)
10th–25th (0.79–1.00)	309 (14)	441 (15)	0.89 (0.75–1.05)	0.95 (0.80–1.14)	0.94 (0.73–1.21)	1.01 (0.77–1.32)
25th–75th (1.00–1.88)	1104 (49)	1440 (50)	1 (reference)	1 (reference)	1 (reference)	1 (reference)
75th–90th (1.88–2.58)	355 (16)	432 (15)	1.09 (0.93–1.29)	1.05 (0.88–1.24)	1.01 (0.80–1.27)	0.98 (0.76–1.26)
>90th (>2.58)	242 (11)	287 (10)	1.13 (0.93–1.36)	0.96 (0.79–1.18)	0.95 (0.72–1.24)	0.89 (0.66–1.20)
Apo B (g L^−1^)						
<10th (<0.68)	292 (13)	285 (10)	1.35 (1.12–1.62)	**1.54 (1.26**–**1.88)**	1.30 (0.99–1.72)	**1.49 (1.10**–**2.00)**
10th–25th (0.68–0.80)	356 (16)	431 (15)	1.10 (0.93–1.29)	**1.20 (1.01**–**1.42)**	1.01 (0.79–1.28)	**1.19 (0.92**–**1.54)**
25th–75th (0.80–1.15)	1071 (48)	1435 (50)	1 (reference)	**1 (reference)**	1 (reference)	**1 (reference)**
75th–90th (1.15–1.33)	313 (14)	425 (15)	1.00 (0.85–1.18)	**0.91 (0.76**–**1.08)**	0.95 (0.74–1.21)	**1.06 (0.81**–**1.38)**
>90th (>1.33)	202 (9)	297 (10)	0.92 (0.75–1.12)	**0.84 (0.68**–**1.03)**	0.98 (0.73–1.33)	**1.01 (0.73**–**1.40)**
HDL-C (mmol L^−1^)						
<10th (<0.90)	272 (12)	289 (10)	1.35 (1.11–1.62)	1.12 (0.91–1.37)	1.04 (0.79–1.38)	0.90 (0.66–1.23)
10th–25th (0.90–1.07)	387 (17)	434 (15)	1.26 (1.07–1.48)	1.17 (0.99–1.39)	1.08 (0.85–1.36)	1.04 (0.81–1.34)
25th–75th (1.07–1.56)	1068 (48)	1449 (50)	1 (reference)	1 (reference)	1 (reference)	1 (reference)
75th–90th (1.56–1.86)	330 (15)	421 (15)	1.03 (0.87–1.22)	1.12 (0.94–1.33)	1.12 (0.87–1.44)	1.14 (0.86–1.49)
>90th (>1.86)	177 (8)	280 (10)	0.82 (0.67–1.01)	0.97 (0.78-1.21)	0.79 (0.58-1.09)	0.80 (0.57-1.13)
Apo A1 (g L^−1^)						
<10th (<1.09)	322 (14)	295 (10)	1.50 (1.25–1.79)	**1.36 (1.12**–**1.64)**	1.26 (0.97–1.65)	**1.19 (0.89**–**1.60)**
10th–25th (1.09–1.22)	347 (16)	429 (15)	1.09 (0.93–1.29)	**1.02 (0.86**–**1.21)**	0.98 (0.77–1.24)	**0.91 (0.70**–**1.18)**
25th–75th (1.22–1.59)	1107 (50)	1444 (50)	1 (reference)	**1 (reference)**	1 (reference)	**1 (reference)**
75th–90th (1.59–1.81)	296 (13)	432 (15)	0.86 (0.73–1.02)	**0.96 (0.80**–**1.15)**	0.84 (0.66–1.08)	**0.91 (0.69**–**1.19)**
>90th (>1.81)	162 (7)	273 (10)	0.73 (0.59–0.90)	**0.88 (0.70**–**1.10)**	0.66 (0.47–0.93)	**0.67 (0.47**–**0.97)**

Data were missing for some participants in some subgroups

*OR* odds ratio, *CI* confidence interval, *TC* total cholesterol, *LDL*-*C* low-density lipoprotein cholesterol, *Apo B* apolipoprotein B, *HDL*-*C* high-density lipoprotein cholesterol, *Apo A1* apolipoprotein A1

^a^Adjusted for age, sex and partnership (where applicable)

^b^Adjusted for age, sex, body mass index, estrogen use at blood sampling, statin use, self-reported diabetes, and partnership (where applicable)

OR^b^ (full adjustment) for apo B and apo A1 is in bold

In analyses using both control groups, decreasing HDL-C and apo A1 levels were associated with an increasing risk of venous thrombosis in a dose-dependent manner (Table 2). Compared with the reference categories, the lowest percentile categories of HDL-C and apo A1 yielded odds ratio for venous thrombosis of 1.35 (95% CI 1.11–1.62) and 1.50 (95% CI 1.25–1.79), respectively. With adjustment for BMI, the risk conferred by apo A1 was attenuated (OR 1.33, 95% CI 1.10–1.60), whereas the risk conferred by HDL-C virtually disappeared (OR 1.10, 95% CI 0.91–1.35). After full adjustment, both risk estimates did not substantially change, and the dose–response relation remained between apo A1 levels and venous thrombosis risk, as depicted in Table 2.

When the analysis was restricted to partners as the control group, there was again no consistent association between venous thrombosis and levels of TC, LDL-C, triglycerides and HDL-C across percentile categories (Table 2). Results for apo B and apo A1 were similar to those obtained with the pooled control group, i.e., risk estimates for venous thrombosis increased with decreasing levels of apo B or apo A1.

As risks of venous thrombosis were apparently not related with TC, LDL-C, triglycerides and HDL-C, these lipids were not further considered in the subsequent analyses.

### Mediation analyses

In comparison with the reference category, an increment in apo B levels across percentile categories was associated with a consistent increase in levels of natural anticoagulants (protein C, protein S, antithrombin, and TFPI), procoagulant factors (fibrinogen, and factors II, VII, IX, X, and XI), clot lysis time, and CRP (Table 3). With the exception of fibrinogen, changes in hemostatic factor levels were in the same direction for apo A1, i.e., as apo A1 increased, levels of protein C, and factors II, VII, IX, X, and XI increased as well (Table 4). Among the procoagulant factors, only factor VIII and von Willebrand factor were not associated with apo B and apo A1. The behavior of the aforementioned hemostatic factor and CRP levels was the same when apo B and apo A1 were introduced continuously in the model.

**Table 3:**
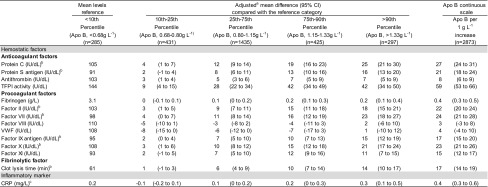
Effect of percentiles of apolipoprotein B on the levels of hemostatic factors and c-reactive protein in control subjects

Data were missing for some participants in some subgroups

*Apo B* apolipoprotein B, *CI* confidence interval, *TFPI* tissue factor pathway inhibitor, *VWF* von Willebrand factor, *CRP* C-reactive protein

^a^Adjusted for age, sex, body mass index, estrogen use at blood sampling, statin use, and self-reported diabetes

^b^Vitamin K antagonists users at the time of blood sampling were excluded from the analyses

^c^Log-transformed

**Table 4:**
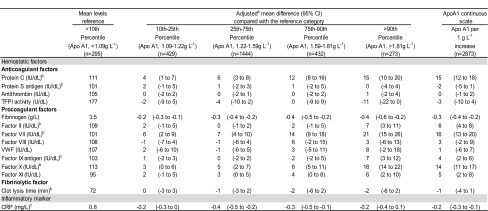
Effect of percentiles of apolipoprotein A1 on the levels of hemostatic factors and c-reactive protein in control subjects

Data were missing for some participants in some subgroups

*Apo A1* apolipoprotein A1*, CI* confidence interval*, TFPI* tissue factor pathway inhibitor, *VWF* von Willebrand factor, CRP *C*-*reactive protein*

^a^ Adjusted for age, sex, body mass index, estrogen use at blood sampling, statin use, and self-reported diabetes

^b^ Vitamin K antagonists users at the time of blood sampling were excluded from the analyses

^c^ Log-transformed

When CRP and the hemostatic factors associated with apo B or apo A1 were added to the logistic regression model (Table 5), risk estimates changed marginally for either apolipoprotein as compared with the analyses adjusted for all potential confounders, regardless the type of control group used. When the potential mediators were entered one by one in the model, results were similar to those described in Table 5 for both apolipoproteins (data not shown).

**Table 5:** Risk of venous thrombosis by percentiles of apolipoproteins B and A1 levels adjusted for hemostatic factors and c-reactive protein

	Pooled control analyses						Partner control analyses					
	OR	(95% CI)^a^	OR	(95% CI)^ab^	OR	(95% CI)^abc^	OR	(95% CI)^a^	OR	(95% CI)^ab^	OR	OR (95% CI)^abc^
Apo B (g L^−1^)												
<10th (<0.68)	1.38	(1.14–1.67)	1.58	(1.28–1.93)	**1.86**	**(1.49–2.31)**	1.29	(0.97–1.71)	1.44	(1.06–2.00)	**1.59**	**(1.12–2.25)**
10th–25th (0.68-0.80)	1.13	(0.95-1.33)	1.22	(1.02–1.45)	**1.36**	**(1.13–1.64)**	1.04	(0.81–1.34)	1.22	(0.93–1.59)	**1.29**	**(0.97–1.72)**
25th–75th (0.80–1.15)	1	(reference)	1	(reference)	**1**	**(reference)**	1	(reference)	1	(reference)	**1**	**(reference)**
75th– 90th (1.15–1.33)	0.98	(0.83–1.17)	0.88	(0.74–1.06)	**0.81**	**(0.67–0.98)**	0.98	(0.76–1.28)	1.09	(0.82–1.45)	**1.01**	**(0.74–1.37)**
>90th (>1.33)	0.80	(0.65–0.99)	0.72	(0.58–0.90)	**0.63**	**(0.49–0.80)**	0.84	(0.60–1.17)	0.88	(0.62–1.26)	**0.88**	**(0.59-1.31)**
Apo A 1 (g L^−1^)												
<10th (< 1.09)	1.42	(1.18–1.72)	1.29	(1.05–1.57)	**1.20**	**(0.98–1.48)**	1.24	(0.93–1.65)	1.17	(0.85–1.59)	**1.09**	**(0.78–1.52)**
10th–25th (1.09–1.22)	1.08	(0.91–1.28)	1.01	(0.84-1.20)	**0.98**	**(0.82–1.18)**	1.03	(0.80–1.33)	0.96	(0.73–1.27)	**0.97**	**(0.73–1.30)**
25th– 75th (1.22-1.59)	1	(reference)	1	(reference)	**1**	**(reference)**	1	(reference)	1	(reference)	**1**	**(reference)**
75th– 90th (1.59–1.81)	0.88	(0.73–1.04)	0.99	(0.83–1.20)	**1.02**	**(0.85–1.24**	0.83	(0.63–1.08)	0.88	(0.66–1.17)	**0.91**	**(0.67–1.22)**
>90th (>1.81)	0.71	(0.56–0.88)	0.86	(0.68–1.09)	**0.84**	**(0.66–1.08)**	0.65	(0.45–0.93)	0.65	(0.44–0.96)	**0.67**	**(0.46–1.00)**

Data were missing for some participants in some subgroups

*OR* odds ratio, *CI* confidence interval, *Apo B* apolipoprotein B, *apo A1* apolipoprotein A1

^a^Adjusted for age, sex and partnership (where applicable)

^b^Adjusted for body mass index, estrogen use at blood sampling, statin use, and self-reported diabetes

^c^Adjusted for potential mediators for apolipoprotein B (protein C, protein S, antithrombin, tissue factor pathway inhibitor, fibrinogen, factors II, VII, IX, X, and XI, clot lysis time, and c-reactive protein) or for apolipoprotein A1 (protein C, fibrinogen, factors II, VII, IX, X, and XI, and c-reactive protein)

OR^a^ and OR^ab^ were slightly different from those depicted in Table 2 because vitamin K antagonist users at the time of blood sampling (n = 271 patients and n = 27 controls) were excluded from the mediation analyses

OR^abc^ (further adjustment for potential mediators) is in bold

## Discussion

In this large population-based case–control study, levels of the major lipids (i.e., TC, LDL-C, triglycerides, or HDL-C) were not associated with an increased risk of venous thrombosis. In contrast, apo B and apo A1 appeared inversely associated with venous thrombosis, as decreasing levels of both apolipoproteins were associated with an increased risk of venous thrombosis, also after adjustment for potential confounders. Although apo B and to a lesser extent apo A1 were associated with several hemostatic factors and CRP, none of these factors explained the association between these apolipoproteins and venous thrombosis risk.

A previous meta-analysis demonstrated that mean levels of triglycerides were higher and those of HDL-C lower in venous thrombosis patients than in controls [6]. However, the majority of the reports on lipids and venous thrombosis in this meta-analysis were small case–control studies, and controlling for several confounders had not been possible [6]. Our results follow the majority of the longitudinal studies published after the aforementioned meta-analysis [6], that collectively showed little to no evidence of an association between the major lipid levels and risk of venous thrombosis [7, 10–12].

In contrast to the major lipids, fewer reports have addressed the relationship between venous thrombosis and levels of apo B and apo A1. In two cohort studies [8, 12] neither apolipoprotein was associated with risk of venous thrombosis. In the other reports, an association between these apolipoproteins and venous thrombosis was restricted to certain subgroups. For instance, in a small case–control study composed of men only, low apo A1 levels were associated with an increased risk of venous thrombosis [29]. On the other hand, high apo A1 levels in the *Women’s Health Study* were associated with an increased thrombosis risk in hormone users [9]. In a hospital-based case–control study, high apo B levels appeared to increase the risk of venous thrombosis mainly in men [13]. Differences related to the study design, the source and selection criteria of the study population, the sample size, and the adjustment for potential confounders might have contributed to the contradictory results among these studies [8, 9, 12, 13, 29].

After excluding a major role for the most important confounders in the association between venous thrombosis and levels of apo B and apo A1, we investigated whether this association might be explained by potential mediators. Although apo B and to a lesser extent apo A1 levels were associated with levels of several hemostatic factors and CRP, their inclusion in the logistic regression model only marginally affected risk estimates conferred by both apolipoproteins, thereby excluding a mediating role for these factors. Nevertheless, the strong association between levels of apolipoproteins and hemostatic factors/CRP deserves some further attention. We can hypothesize that this may reflect a common mechanism, that influences the synthesis of these proteins in hepatocytes [30–32]. In line with this hypothesis was the finding that factor VIII and von Willebrand factor, both largely expressed by endothelial cells [31, 33], were not associated with apo B or apo A1 levels. TFPI fits this hypothesis with respect to apo A1 (no relation found), as it is also primarily synthesized by endothelial cells [34]. The association between total TFPI activity and apo B levels that was nevertheless found is consistent with the fact that the majority of the TFPI in plasma is bound to apo B-containing lipoproteins, mainly LDL [34].

Here we observed a protective role for apo B against venous thrombosis. Conversely, there is strong evidence that elevated apo B and LDL-C are associated with increased risk for arterial disease [35, 36]. Still, one should consider that even though arterial and venous thrombosis share some risk factors [1], their pathophysiology is different. An arterial thrombus is typically formed after rupture of an atherosclerotic plaque, whereas venous thrombi assemble on the surface of a largely intact vessel wall [37]. Based on this, it would be reasonable to consider that apo B may play different roles in the mechanisms that are at the basis of venous versus arterial thrombosis. Indeed, our results on the inverse association of apo B levels with risk of venous thrombosis are consistent with experimental studies demonstrating that apo B is capable of inhibiting coagulation [38, 39]. *In vitro*, purified human apo B was shown to inhibit TF-initiated coagulation either alone or reconstituted into LDL-like particles in a thus far undefined TFPI-independent manner [39]. Alternatively, apo B could indirectly exert an anticoagulant function via LDL-bound TFPI. The latter was shown to inhibit the coagulation protease factor Xa activity more potently as compared with the TF-FVIIa inhibition [40]. In the present study, we assessed total plasma TFPI activity via inhibition of the TF-FVIIa complex, which might explain why TFPI did not mediate, at least in part, the association between apo B and venous thrombosis risk.

It might be argued that our findings on apo B and venous thrombosis are not consistent with the putative protection conferred by statins against venous thrombosis [3–5], since these drugs decrease apo B levels [41, 42]. It is noteworthy that statins may have antithrombotic effects that are unrelated to their lipid-lowering activity, such as downregulation of TF [43]. It is currently unknown, however, to what extent the antithrombotic potential of statins would influence the thrombosis risk or the anticoagulant properties of apo B demonstrated in vitro [38, 39].

Unlike apo B, risk estimates for venous thrombosis associated with apo A1 pointed in the same direction to that reported in observational studies on arterial disease [36]. Importantly, our results are consistent with experimental data on the role of this apolipoprotein in protecting mice against venous thrombosis [44]. It has been demonstrated that flow restriction-induced venous thrombosis was more common in apo A1^−/−^ mice as compared with wild-type mice, and intravenous infusion of human apo A1 prevented venous thrombosis in wild-type mice but not in mice lacking scavenger receptor B type I or endothelial nitric oxide synthase [44]. The interaction between apo A1 within HDL-C and scavenger receptor B type I promotes not only the reverse cholesterol transport but also has been shown to stimulate the activation of endothelial nitric oxide synthase [45], which results in the production of nitric oxide. An impairment in nitric oxide bioavailability represents a central feature of endothelial cell activation and dysfunction [46]. As endothelial activation could contribute to venous thrombus formation [37], according to the the murine model study [44] one of the possible mechanisms by which apo A1 may protect against venous thrombosis is the upregulation of nitric oxide production and as a result the maintenance of endothelial integrity. Additionally, apo A1 has been suggested to have anticoagulant properties in vitro, as apo A1 was observed to play a critical role in the ability of HDL to enhance the anticoagulant potential of the protein C pathway [21, 47].

The strengths of this study include the large patient sample and the detailed assessment of the relationship between lipids and venous thrombosis, considering not only confounding by several common risk factors, but also mediation via hemostatic factors. Estimation of venous thrombosis risk using only partners as controls enabled us to adjust for further confounding by socio-economic factors [28]. Importantly, variation in lipid levels according to demographic and clinical characteristics in controls (age, sex, BMI, and estrogen- and statin-use) agreed with previous findings [2, 14, 15, 18, 19]. Furthermore, our results contributed to clarify the protective role of apo A1 against venous thrombosis, confirming prior findings from a case–control study [29] and a murine model of venous thrombosis [44]. Our study is the first, to our knowledge, to show that apo B levels are also inversely associated with risk of venous thrombosis, a finding supported by the anticoagulant properties of this apolipoprotein observed in vitro [38, 39].

Since this is an etiologic study, alternative explanations for our results should be addressed and considered as potential limitations. First, we cannot rule out that chance has played a role in our results. Still, chance seems an unlikely explanation owing to the dose–response association between the risk of venous thrombosis and levels of apo B and apo A1 in most of our models. Second, one could argue that sampling of the blood after the event of venous thrombosis might have resulted in reverse causation, i.e., the venous thrombotic event has led to changes in lipid levels. Indeed, after venous thrombosis, patients may have modified certain lifestyle factors, which could have affected their lipid profile. Randomized clinical trials published during the nineties showed that changes in diet affected lipid levels in individuals with dyslipidemia [48, 49]. However, the reduction in the levels of TC, LDL-C and apo B produced by a low-fat diet taken from 9 weeks to 1 year in these trials was small or virtually absent without concomitant statin use or aerobic-exercise program [48, 49]. It is quite unlikely that venous thrombosis patients would systematically adhere to a low-fat diet in addition to statin use and/or exercise after the thrombotic event, especially considering that such recommendation is not routinely given, in contrast to arterial disease. Another mechanism for reverse causation would be the effect of acute phase reactions brought about by the thrombotic event on lipid levels [50, 51]. During acute phase reactions in humans, triglyceride levels typically increase and HDL-C and apo A1 levels decrease, whereas TC, LDl-C and apo B levels can either decrease or do not change [50, 51]. Nevertheless, this is an unlikely mechanism for reverse causation, since blood was collected with a median of 10 months after the thrombotic event, by which time the effects of the acute-phase reaction will have worn off [52, 53]. Third, low cholesterol levels have been reported in some chronic illnesses, such as malignancy, rheumatic disorders, hyperthyroidism and tuberculosis [54]. Hence, these illnesses could have been confounding factors but subjects with malignancy were excluded from the current analyses. Furthermore, changes in lipid levels in our study population owing to reverse causation or chronic illnesses would be expected to affect not only the direction of the point estimates related to the apolipoproteins but also to the other lipids, such as TC, which also makes it unlikely that our findings can be due to either of these explanations. Fourth, even though it is reasonable to assume that patients and their partners likely have a similar lifestyle, lipid levels could be influenced by factors that couples may not share, such as physical activity or diet. Thereby, we cannot rule out the possibility of residual lifestyle-related confounders. However, to reduce this possibility, in the conditional regression analyses we also included in our models BMI, statin use, and diabetes as potential sources of confounding related to lifestyle. Finally, LDL-C was not assessed directly but estimated by the Friedewald formula [25]. From an analytical perspective, estimation of LDL-C was the sum of the inaccuracies and imprecision of three measurements [TC, HDL-C and triglyceride], whereas apo B was measured by a direct and standardized method [23, 55].

In conclusion, in this large, unselected population we showed no association between levels of TC, LDL-C, HDL-C and triglycerides and risk of venous thrombosis. Decreasing levels of apolipoproteins B and A1 were associated with increased risk of venous thrombosis, which we could not explain through several proposed mechanisms, such as confounding or other non-causal explanations, or through mediation via inflammation or changes in the hemostatic factors. Our results may form the basis for further studies to confirm these findings, as well as to assess the physiological relevance of the anticoagulant properties of apo B and apo A1, and to determine the mechanism, whether or not causal, underlying the link between these apolipoproteins and venous thrombosis.

## Electronic supplementary material

Below is the link to the electronic supplementary material.

Supplementary material 1 (DOCX 119 kb)
