# Supplementary material for: Lipid levels and risk of venous thrombosis: results from the MEGA-study
Source: Eur J Epidemiol. 2017 May 24;32(8):669–81. doi: 10.1007/s10654-017-0251-1 (PMC5591362; doi:10.1007/s10654-017-0251-1)
Supplement: Supplementary file 1 [file 10654_2017_251_MOESM1_ESM.docx]

Supplementary Table 1 Baseline characteristics

	Patients		Partners		RDD		Partners + RDD	
	MEGA<br>n = 4463	Tested<br>n = 2234	MEGA<br>n = 3222	Tested<br>n = 1455	MEGA<br>n = 2939	Tested<br>n = 1418	MEGA<br>n = 6161	Tested<br>n = 2873
Age, years	49 (25-68)	49 (26-67)	50 (28-66)	51 (29-66)	45 (23-67)	48 (24-67)	47 (25-66)	50 (27-67)
Women, n (%)	2442 (55)	1217 (55)	1626 (51)	746 (51)	1682 (57)	763 (54)	3308 (54)	1509 (53)
BMI (Kg/m^2)	26 (20-36)	26 (20-35)	25 (20-34)	26 (20-33)	25 (20-33)	25 (20-32)	25 (20-33)	25 (20-33)
Estrogen use, n (% in women)								
Index date	1355 (57)	726 (61)	379 (24)	179 (25)	548 (34)	227 (30)	927 (29)	406 (28)
Blood sampling	NA	205 (18)	NA	159 (23)	NA	222 (30)	NA	381 (26)
Statin use, n (%)	139 (3)	72 (3)	178 (6)	75 (5)	167 (6)	108 (8)	345 (6)	183 (6)
Self-reported diabetes, n (%)	152 (4)	72 (3)	101 (3)	46 (3)	85 (3)	41 (3)	186 (3)	87 (3)
Venous thrombosis								
DVT, n (%)	2613 (59)	1307 (59)	NA	NA	NA	NA	NA	NA
PE ± DVT, n (%)	1850 (41)	927 (41)	NA	NA	NA	NA	NA	NA

Continuous variables are shown as median (5th percentile - 95th percentile) and categorical variables as number (%)

Data were missing for some participants in some subgroups

MEGA Multiple Environmental and Genetic Assessment of risk factors for venous thrombosis study, *RDD* random-digit dialing, *BMI* body mass index, *DVT* deep vein thrombosis, *PE* pulmonary embolism, *NA* not applicable

Supplementary Table 2 Lipid levels according to demographic and clinical characteristics in controls

	Controls (n)	Mean level	Mean difference (95% CI)[a]	Mean difference (95% CI)[b]
TC		(mmol L^{-1})		
Age, years				
18 - 50	1474	5.31	reference	reference
50 - 70	1399	5.93	0.62 (0.54 to 0.70)	0.64 (0.55 to 0.72)
Sex				
Men	1364	5.59	reference	reference
Women	1509	5.63	0.05 (-0.03 to 0.12)	0.03 (-0.05 to 0.12)
Body mass index				
Normal weight	1332	5.47	reference	reference
overweight	1078	5.79	0.20 (0.12 to 0.29)	0.21 (0.13 to 0.29)
obesity	357	5.64	0.01 (-0.06 to 0.07)	0.05 (-0.01 to 0.11)
Self-reported diabetes				
No	2774	5.62	reference	reference
Yes	87	5.24	-0.65 (-0.87 to -0.42)	-0.51 (-0.74 to -0.28)
Estrogen use (women)				
Nonusers	1064	5.70	reference	reference
Users	381	5.37	0.03 (-0.09 to 0.15)	0.04 (-0.09 to 0.16)
Statin use				
Nonusers	2678	5.63	reference	reference
Users	183	5.27	-0.72 (-0.88 to -0.56)	-0.66 (-0.82 to -0.49)
LDL-C		(mmol L^{-1})		
Age, years				
18 - 50	1457	3.33	reference	reference
50 - 70	1371	3.79	0.46 (0.39 to 0.53)	0.47 (0.40 to 0.54)
Sex				
Men	1326	3.63	reference	reference
Women	1502	3.48	-0.14 (-0.20 to -0.07)	-0.13 (-0.20 to -0.05)
Body mass index				
Normal weight	1326	3.43	reference	reference
overweight	1050	3.72	0.19 (0.12 to 0.26)	0.19 (0.12 to 0.27)
obesity	347	3.59	0.02 (-0.03 to 0.07)	0.05 (0 to 0.11)
Self-reported diabetes				
No	2732	3.56	reference	reference
Yes	84	3.18	-0.59 (-0.78 to -0.39)	-0.45 (-0.65 to -0.25)
Estrogen use (women)				
Nonusers	1059	3.56	reference	reference
Users	380	3.22	-0.06 (-0.17 to 0.05)	-0.04 (-0.15 to 0.07)
Statin use				
Nonusers	2639	3.58	reference	reference
Users	177	3.16	-0.72 (-0.86 to -0.59)	-0.67 (-0.81 to -0.53)
Triglycerides[c]		(mmol L^{-1})		
Age, years				
18 - 50	1474	0.26	reference	reference
50 - 70	1399	0.41	0.15 (0.11 to 0.18)	0.10 (0.07 to 0.14)
Sex				
Men	1364	0.42	reference	reference
Women	1509	0.25	-0.17 (-0.20 to -0.14)	-0.19 (-0.22 to -0.15)
Body mass index				
Normal weight	1332	0.18	reference	reference
overweight	1078	0.46	0.24 (0.21 to 0.28)	0.24 (0.20 to 0.27)
obesity	357	0.53	0.16 (0.13 to 0.18)	0.16 (0.13 to 0.18)
Self-reported diabetes				
No	2774	0.32	reference	reference
Yes	87	0.66	0.27 (0.17 to 0.36)	0.16 (0.06 to 0.26)
Estrogen use (women)				
Nonusers	1064	0.24	reference	reference
Users	381	0.28	0.13 (0.08 to 0.18)	0.15 (0.10 to 0.21)
Statin use				
Nonusers	2678	0.32	reference	reference
Users	183	0.55	0.13 (0.06 to 0.20)	0.09 (0.02 to 0.16)

Supplementary Table 2 Continued

	Controls (n)	Mean level	Mean difference (95% CI)[a]	Mean difference (95% CI)[b]
Apo B		(g L^{-1})		
Age, years				
18 - 50	1474	0.93	reference	reference
50 - 70	1399	1.05	0.12 (0.10 to 0.14)	0.12 (0.10 to 0.13)
Sex				
Men	1364	1.02	reference	reference
Women	1509	0.95	-0.07 (-0.09 to -0.06)	-0.08 (-0.10 to -0.06)
Body mass index				
Normal weight	1332	0.93	reference	reference
overweight	1078	1.04	0.07 (0.05 to 0.09)	0.08 (0.05 to 0.10)
obesity	357	1.03	0.03 (0.02 to 0.05)	0.04 (0.02 to 0.05)
Self-reported diabetes				
No	2774	0.99	reference	reference
Yes	87	0.97	-0.07 (-0.13 to -0.02)	-0.07 (-0.12 to -0.01)
Estrogen use (women)				
Nonusers	1064	0.96	reference	reference
Users	381	0.93	0.04 (0.01 to 0.07)	0.05 (0.02 to 0.08)
Statin use				
Nonusers	2678	0.99	reference	reference
Users	183	0.96	-0.11 (-0.15 to - 0.07)	-0.11 (-0.14 to -0.07)
HDL-C		(mmol L^{-1})		
Age, years				
18 - 50	1474	1.32	reference	reference
50 - 70	1399	1.37	0.06 (0.03 to 0.08)	0.10 (0.07 to 0.12)
Sex				
Men	1364	1.18	reference	reference
Women	1509	1.50	0.32 (0.29 to 0.34)	0.30 (0.27 to 0.32)
Body mass index				
Normal weight	1332	1.45	reference	reference
overweight	1078	1.26	-0.17 (-0.19 to -0.14)	-0.16 (-0.19 to -0.13)
obesity	357	1.18	-0.14 (-0.16 to -0.12)	-0.13 (-0.15 to -0.11)
Self-reported diabetes				
No	2774	1.35	reference	reference
Yes	87	1.13	-0.23 (-0.31 to -0.16)	-0.15 (-0.23 to -0.08)
Estrogen use (women)				
Nonusers	1064	1.50	reference	reference
Users	381	1.51	0.03 (-0.02 to 0.08)	0.01 (-0.04 to 0.06)
Statin use				
Nonusers	2678	1.35	reference	reference
Users	183	1.26	-0.08 (-0.13 to -0.02)	-0.04 (-0.09 to 0.02)
apo A1		(g L^{-1})		
Age, years				
18 - 50	1474	1.40	reference	reference
50 - 70	1399	1.46	0.06 (0.04 to 0.08)	0.09 (0.07 to 0.11)
Sex				
Men	1364	1.30	reference	reference
Women	1509	1.54	0.24 (0.22 to 0.26)	0.20 (0.18 to 0.22)
Body mass index				
Normal weight	1332	1.48	reference	reference
overweight	1078	1.38	-0.08 (-0.10 to -0.06)	-0.07 (-0.09 to -0.05)
obesity	357	1.35	-0.07 (-0.09 to -0.05)	-0.06 (-0.08 to -0.05)
Self-reported diabetes				
No	2774	1.43	reference	reference
Yes	87	1.33	-0.12 (-0.18 to -0.07)	-0.09 (-0.14 to -0.03)
Estrogen use (women)				
Nonusers	1064	1.52	reference	reference
Users	381	1.61	0.13 (0.09 to 0.16)	0.12 (0.08 to 0.15)
Statin use				
Nonusers	2678	1.43	reference	reference
Users	183	1.42	-0.01 (-0.05 to 0.03)	0.01 (-0.03 to 0.05)

Data were missing for some participants in some subgroups
Estrogen use was determined at the time of blood sampling
Normal weight was defined as a body mass index 18.5-25 kg/m^2, overweight as a body mass index 25-30 kg/m^2, and obesity as a body mass index >30 kg/m^2
TC total cholesterol, *LDL-C* low-density lipoprotein cholesterol, *Apo B* apolipoprotein B, *HDL-C* high-density lipoprotein cholesterol, *Apo A1* apolipoprotein A1
[a]Mean difference adjusted for age and sex (where applicable)
[b]Mean difference adjusted for age, sex, body mass index, self-reported diabetes, estrogen use at blood sampling, and statin use (where applicable)
[c]Log-transformed
